# Correlation Between Serum miR-154-5p and Osteocalcin in Males and Postmenopausal Females of Type 2 Diabetes With Different Urinary Albumin Creatinine Ratios

**DOI:** 10.3389/fendo.2019.00542

**Published:** 2019-08-06

**Authors:** Huiwen Ren, Xiaoyu Ma, Ying Shao, Jinyu Han, Min Yang, Qiuyue Wang

**Affiliations:** ^1^Department of Endocrinology, The First Hospital Affiliated of China Medical University, Shenyang, China; ^2^The Cadre Department, The First Hospital of China Medical University, Shenyang, China; ^3^Department of Endocrinology, The Second Hospital Affiliated of China Medical University, Shenyang, China; ^4^Department of Laboratory Medicine, The First Hospital Affiliated of China Medical University, Shenyang, China

**Keywords:** type 2 diabetes mellitus, miR-154-5p, osteocalcin, diabetic kidney disease, diabetic osteoporosis

## Abstract

**Purpose:** To investigate the serum levels of miR-154-5p, osteocalcin (OC), and other clinical parameters in male and post-menopausal female type 2 diabetes mellitus (T2DM) patients with different urinary albumin creatinine ratio (UACR) levels and to discuss the relationship between miR-154-5p and glycolipid metabolism, bone metabolism, and different urinary albumin excretion rate in T2DM.

**Methods:** Seven hundred thirty-eight T2DM patients were categorized into six groups, including 374 men and 364 post-menopausal women who were sub-divided into three groups based on albumin excretion that involved normal albuminuria, microalbuminuria, and large amount of albuminuria (138, 127, 109, 135, 125, and 104 cases, UACR<30, 30–300, and >300 mg/g, M1, M2, M3, F1, F2, and F3). Measurement of circulating miR-154-5p, OC, and other biochemical indicators were performed by real-time PCR, ELISA, and chemiluminescence assays in T2DM patients and in 141 M0 and 139 F0 control subjects.

**Results:** There are few differences appeared between groups. Comparing with men, women had higher age, waist-to-hip ratio (WHR), adiponectin (ADPN), connective tissue growth factor (CTGF), UACR, procollagen type 1 N-terminal propeptide (P1NP), β-C-terminal telopeptide of type I collagen (β-CTx), OC, and miR-154-5p, but lower FPG, HOMA-IR, and HbA1c. T2DM patients with albuminuria (micro or macro) had lower bone turnover markers (P1NP, β-CTx, and OC) and adiponectin, but higher HbA1c, CTGF, and miR-154-5p. In addition, after regression analysis, UACR was positively correlated with CTGF, HbA1c, and miR-154-5p, and negatively correlated with ADPN and bone turnover markers (P1NP, β-CTx, and OC). However, OC showed a positive correlation with ADPN and other bone turnover markers (P1NP and β-CTx), but negative correlation with CTGF, UACR, and miR-154-5p in all three groups.

**Conclusion:** These findings suggested that increased serum levels of miR-154-5p and decreased OC levels may influence osteogenesis and proteinuria in T2DM and may identify novel targets for diagnosis and treatment of diabetic kidney disease and osteoporosis.

## Highlights

- Serum miR-154-5p was elevated in post-menopausal female T2DM patients and increased successively with UACR.
- Serum miR-154-5p was found to positively correlate with UACR and negatively correlate with OC.
- OC and UACR were associated with glycolipid metabolism, bone metabolism, and urinary albumin.

## Introduction

Type 2 diabetes mellitus (T2DM), recognized as a global epidemic by the World Health Organization, is a long-term metabolic disorder characterized by high blood glucose, insulin resistance, and relative lack of insulin. One of the chronic complications of T2DM is diabetic kidney disease (DKD) that causes glomerular fibrosis, persistent proteinuria, and eventually, end-stage renal disease (ESRD) (1, 2). Another chronic complication of T2DM is diabetic osteoporosis (DOP). Clinical evidences indicate that the risk of low bone mass, osteoporosis, and fracture increases in T2DM, especially in elder men and post-menopausal women (3–7). The pathogenesis of these diseases has not been fully elucidated and is considered related to renal dysfunction and abnormal bone metabolism caused by glucolipid metabolism-related disorders (1–7).

The balance between osteoblasts and osteoclasts activities determines bone mass and osteocytes orchestrate these communications in reaction to endocrine and mechanical stimuli. Osteocalcin (OC), procollagen type 1 N-terminal propeptide (P1NP), and alkaline phosphatase (ALP) are the markers of bone formation, while β-C-terminal telopeptide of type I collagen (β-CTx) is the characteristic biochemical indicator of bone resorption. Moreover, 25(OH) Vitamin D3 [25(OH) VD3] and parathyroid hormone (PTH) promote absorption of calcium and phosphorus and increase blood calcium (Ca) and phosphorus (P) concentration thereby promoting bone calcification (8, 9). Previous studies have reported that these bone metabolic indicators may be involved in the crosstalk between bone, islet, and adipose tissues (8, 10–13) and may be associated with early renal damage in DKD (14, 15). However, these findings remain controversial and inconclusive.

MiR-154, a recently discovered microRNA with anti-cancer effects, is involved in pathophysiology of various disorders (16–20). It has been reported that miR-154-5p, a mature serum sequence of miR-154 (21–23), regulates osteogenic and differentiation of mesenchymal stem cells derived from adipose (24) and may be involved in renal fibrosis (25–27). These data suggest a potential association between miR-154-5p and pathology of osteogenic differentiation and urinary protein increasing in chronic kidney diseases.

We investigated the relationship between serum levels of miR-154-5p and other clinical parameters such as glucolipid metabolism and bone metabolism in male and post-menopausal female T2DM patients with different UACR levels.

## Materials and Methods

### Subjects

We conducted a cross-sectional cohort study of 738 T2DM patients initially diagnosed and treated at the Department of Endocrinology of the First Hospital Affiliated of China Medical University from August 2017 to December 2018. The normal control groups (141 and 139 cases for M0 and F0, respectively) were from the Health Examination Center of the First Hospital Affiliated to China Medical University. This study was approved by the Ethics Committee of China Medical University and all participants signed informed consent.

Inclusion criteria included: men diagnosed with T2DM based on ADA standard (26) and post-menopausal women diagnosed with both T2DM and spontaneous menstruation for more than 1 year. Exclusion criteria included: (1) diabetic hyperosmolar coma, diabetic ketoacidosis or other acute diabetic complications in the last 3 months; (2) cerebrovascular diseases, liver function damage, infectious diseases, hemolysis induced by related diseases, or a history of malignant tumor; (3) recent stress, such as infection, surgery, trauma, or special physical conditions, such as pregnancy and lactation; (4) patients using angiotensin-converting enzyme inhibitors (ACEI) or angiotensin-receptor blockers (ARB) to affect the excretion rate of urinary albumin; (5) history of fracture within 1 year, osteoporosis, thyroid, parathyroid diseases, or other endocrine diseases; (6) use of agents that may affect bone metabolism such as thiazolidinediones, vitamin K, warfarin, vitamin D, calcium supplement, bisphosphonates, estrogen, and hormones, and agents that may lower lipid levels.

The subjects included 374 males and 364 post-menopausal females with T2DM. All the subjects were divided into six groups among males and post-menopausal females: (1) normal albuminuria male group (138 cases, UACR <30 mg/g, M1), (2) microalbuminuria male group (127 cases, UACR 30–300 mg/g, M2), (3) large amount of albuminuria male group (109 cases, UACR>300 mg/g, M3); (4) normal albuminuria post-menopausal female group (135 cases, UACR <30 mg/g, F1), (5) microalbuminuria post-menopausal female group (125 cases, UACR 30–300 mg/g, F2), and (6) large amount of albuminuria post-menopausal female group (104 cases, UACR>300 mg/g, F3) based on urinary albumin excretion rate (UACR) (27). Meanwhile, the age-matched normal control male and post-menopausal female groups (141 and 139 cases for M0 and F0, respectively) were included from physical examination center during the same period.

Fasting blood samples were collected from T2DM patients using standard venipuncture (28) including non-hemolized samples with an absorbance <0.2 at 414 nm (29). Two samples of 5 ml venous blood were collected without anticoagulant and kept at room temperature for 30 min. The first sample was centrifuged for 15 min (1,000 × g, 4°C) for enzyme-linked immunosorbent assay (ELISA) and the second sample was centrifuged for 5 min (2,000 × g, 4°C) for real-time PCR assay as we conducted in the previous research (30). The serum samples were collected, sealed, and stored at −80°C till further use.

### Measurements

All volunteers were asked about age, course of T2DM and menstrual history. The height (H), weight (W), waist circumference, hip circumference, and blood pressure (SBP, DBP) were monitored according to a standard protocol (31). Body mass index (BMI) was calculated as H (m)^2^/W (kg) while waist hip rate (WHR) was calculated as waist circumference (cm)/hip circumference (cm). Fasting blood samples by standard venipuncture and moderate morning urine were collected from all subjects. Double centrifugation with a Beckman J-6M Induction Drive Centrifuge (Beckman Coulter, Inc., Brea, CA, USA) was used to separate the serum. All urine, serum, and plasma samples were stored at −80°C until final analysis.

Sandwich ELISA was used to measure adiponectin (ADPN, product number: CSB-E07270h; detection range: 1.56 −100 ng/ml; CUSABIO, Wuhan, P.R.China) and connective tissue growth factor (CTGF, product number: CSB-E07875h; detection range: 3.75–120 pg/ml; CUSABIO, Wuhan, P.R.China) in the serum samples according to the manufacturers' instructions. Inter- and intra-assay coefficients of variation was between <8 and <10%, respectively.

Glucose oxidase examined by double-antibody radioimmunoassay were used to measure fasting plasma glucose (FPG), fasting plasma insulin (FINS), and fasting c-peptide (FCP) levels. The homeostasis model assessment of insulin resistance (HOMA-IR) was used to estimate insulin resistance as follows: FINS (mU/L) × FPG (mmol/L)/22.5 and insulin sensitive index (ISI = −ln[FPG × FINS]). Glycosylated hemoglobin (HbA1c) was detected using an automated HbA1c analyzer (Bio-Rad, Hercules, CA, USA).

An automated biochemical analyzer (Beckman Coulter, Inc., Brea, CA, USA) was used to measure urinary albumin, urinary creatinine (uCr), total triglyceride (TG), total cholesterol (TC), low-density lipoprotein cholesterol (LDL-C), high-density lipoprotein cholesterol (HDL-C), Ca, P, PTH, 25(OH)VD3, ALP, OC, P1NP, as well as β-CTx in the Laboratory Medicine, Nephrology Laboratory, Central Laboratory and the Laboratory of Endocrine and Metabolism of the First Hospital of China Medical University. UACR (urinary albumin/creatinine ratio) was calculated to estimate the levels of urinary proteins (32, 33).

### MiRNA Isolation and Quantitative PCR Analysis

MiRNA was extracted from 400 μL serum samples using a miRcute miRNA isolation kit (DP501, Beijing Tiangen) (34). Before extraction, the *C. elegans* synthetic miRNA- cel-miR-39- mimic (Qiagen, Hilden, Germany) was added to each serum sample to correct for extraction errors. Strand of cDNA modified by tail reverse transcription was synthesized using a miRcute miRNA First-Strand cDNA Synthesis Kit (Tiangen, Beijing, China) (35). Real-time PCR detection of miR-154-5p in 3.0 μL cDNA template was performed using a miRcute miRNA qPCR Detection Kit (FP401, SYBR Green, Tiangen, Beijing, China) on Takara Thermal Cycler Dice Real Time System. PCR amplification was performed using the following conditions: initial denaturation at 94°C for 2 min, 45 cycles denaturation at 95°C for 5 s, annealing and extension at 60°C for 40 s. Absolute quantification of miR-154-5p in each sample was calculated using the 2∧ (-ΔCt) method (26, 36).

### Statistical Analysis

IBM SPSS Statistics (V.22.0, IBM Corp., Armonk, NY, USA) was used for data analysis. The normality of each group was judged according to the previous literature and normality test. All values were expressed as the mean ± SD for normally distributed values and as median (interquartile range) for non-parametric values. Difference between UACR groups was analyzed by one-way ANOVA and sex groups by Student's *t*-test for normally distributed values and Kruskal-Wallis *H*-test for non-parametric values. The least-significant difference *t*-test or Mann-Whitney *U*-test were used for pairwise comparison of differences among multiple groups. After logarithmic transformation was used for non-parametric values, clinical parameters related to OC and Ln UACR (Natural logarithmic UACR) were analyzed using Pearson correlation and multiple linear regression analysis. When there was collinearity, ridge regression analysis was performed to determine the association between the clinical parameters. All *P*-values were two-tailed, and *P* < 0.05 was considered statistically significant.

## Results

### Serum miR-154-5p Levels and Clinical Characteristic in the Male and Post-menopausal Female Groups

**Figure 1:**
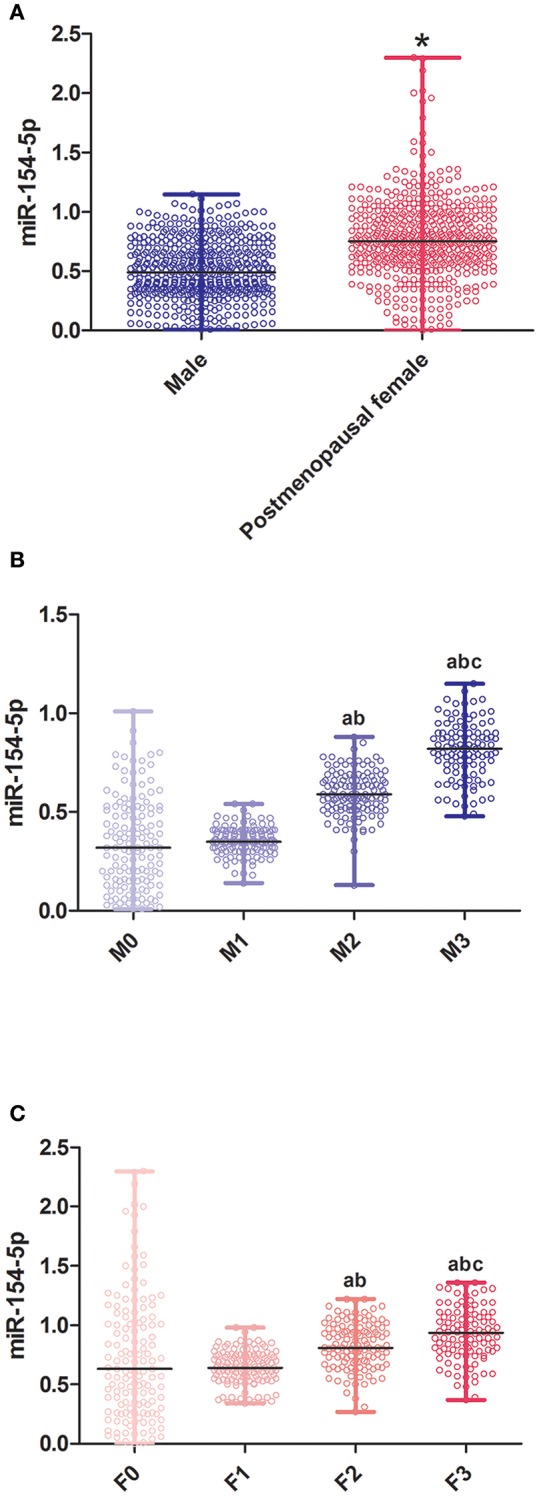
Relative expression of miR-154-5p in different sex and UACR groups. **(A)** Between genders, ^*^post-menopausal female vs. Male, *P* < 0.05; **(B)** different UACR in male patients, a F1-3 vs. F0, *P* < 0.05, b F3, F2 vs. F1, *P* < 0.05, c F3 vs. F2, *P* < 0.05; and **(C)** different UACR in post-menopausal female patients, a M1-3 vs. M0, *P* < 0.05, b M3, M2 vs. M1, *P* < 0.05, c M3 vs. M2, *P* < 0.05. Data were expressed by median (quartile). M0, the normal control male group; M1-3, the normal albuminuria, microalbuminuria, and large amount of albuminuria male groups; F0, the normal control post-menopausal female group; F1-3, the normal albuminuria, microalbuminuria, and large amount of albuminuria post-menopausal female groups; UACR, urinary albumin excretion rate.

1018 volunteers participated in our study, included 515 males and 503 post-menopausal females. We observed significant differences in age and levels of WHR, FPG, HOMA-IR, HbA1c, ADPN, P1NP, β-CTx, OC, CTGF, UACR, and miR-154-5p (*P* < 0.05, Table 1 and Figure 1A) between the two groups. Compared to the male group, age, WHR, ADPN, P1NP, β-CTx, OC, CTGF, UACR, and miR-154-5p were elevated and FPG, HOMA-IR, and HbA1c were significantly reduced in the post-menopausal female group (*P* < 0.05). However, no significant difference was observed in other parameters between different genders (*P* > 0.05).

**Table 1:** Levels of serum miR-154-5p and clinical characteristic in the studied groups.

	**Male**	**Post-menopausal female**	***T/Z***	***P-*values**
*N*	515	503	–	–
Age (years)	53.84 ± 10.20	63.84 ± 10.60^*^	−15.3464	<0.0001
Course (months)	87 (0–96)	86 (0–97)	−0.3363	0.7366
BMI (kg/m^2^)	24.78 ± 2.84	24.56 ± 2.61	1.2755	0.2024
WHR	0.92 ± 0.06	0.93 ± 0.06^*^	−2.0238	0.0433
FPG (mmol/L)	8.68 ± 2.95	7.66 ± 2.58^*^	5.8227	<0.0001
FINS (mIU/L)	11.87 ± 6.69	11.83 ± 6.42	0.1056	0.9159
FCP (pmol/L)	7.99 ± 2.41	8.27 ± 2.49	−1.7958	0.0728
ISI	−3.84 ± 1.38	−3.79 ± 1.27	−0.5588	0.5765
HOMA-IR	4.69 ± 3.55	4.14 ± 2.99^*^	2.6658	0.0078
HbA1c (%)	8.1 (5.8–9.5)	7.5 (4.9–8.5)^*^	−6.9392	<0.0001
HDL-C (mmol/L)	1.07 ± 0.25	1.08 ± 0.26	−0.9609	0.3368
LDL-C (mmol/L)	3.24 ± 0.94	3.20 ± 0.89	0.6683	0.5041
TC (mmol/L)	5.00 ± 1.21	4.99 ± 1.24	0.2341	0.8149
TG (mmol/L)	2.23 ± 1.00	2.12 ± 0.94	1.7688	0.0772
ADPN (mg/L)	25.57 ± 6.44	32.39 ± 6.36^*^	−16.9782	<0.0001
25(OH)VD3(pg/ml)	7.95 (6.65–8.82)	7.93 (6.77–9.10)	−1.1214	0.2621
PTH (pg/ml)	34.18 (30.83–37.54)	33.78 (30.33–36.64)	−1.1481	0.2509
P1NP (pg/ml)	33.43 ± 10.35	48.61 ± 14.88^*^	−18.9326	<0.0001
β-CTx (pg/ml)	384.24 ± 77.92	525.02 ± 130.74^*^	−20.9261	<0.0001
Ca (mmol/L)	2.23 ± 0.15	2.23 ± 0.15	−0.0251	0.9799
P (mmol/L)	1.16 ± 0.20	1.18 ± 0.19	−1.7010	0.0892
ALP (U/L)	70.94 ± 20.72	69.55 ± 19.37	1.1068	0.2686
OC (pg/ml)	14.31 ± 5.59	20.20 ± 8.76^*^	−12.8062	<0.0001
CTGF (pg/ml)	246.16 ± 96.31	297.55 ± 134.78^*^	−7.0125	<0.0001
UACR (mg/g)	17.22 (13.84–116.73)	19.15 (15.90–116.95)^*^	−3.9030	0.0001
miR-154-5p	0.49 (0.35–0.68)	0.75 (0.58–0.95)^*^	−13.4884	<0.0001

*Data are expressed as the mean ± SD or the median (interquartile range). BMI, body mass index; WHR, waist hip rate; FPG, fasting plasma glucose; FINS, fasting insulin; FCP, fasting c-peptide; ISI, insulin sensitive index (ISI = –ln[FPG × FINS]); HOMA-IR, homeostatic model assessment of insulin resistance (HOMA-IR = FPG × FINS/22.5); HbA1c, glycated hemoglobin A1c; HDL-C, high-density lipoprotein cholesterol; LDL-C, low-density lipoprotein cholesterol; TC, total cholesterol; TG, triglyceride; ADPN, adiponectin; PTH, parathyroid hormone; P1NP, procollagen type 1 N-terminal propeptide; β-CTx, β-C-terminal telopeptide of type I collagen; Ca, calcium; P, phosphorus; ALP, alkaline phosphatase; OC, osteocalcin; CTGF, connective tissue growth factor; UACR, urinary albumin excretion rate. Post-menopausal female vs. male*,

* *P <0.05*.

### Serum miR-154-5p Levels and Clinical Characteristic in Male T2DM Groups With Different UACR

Table 2 and Figure 1B illustrates the changes in serum biological markers in male T2DM patients with different UACR. Compared to the M0 group, course, FPG, FINS, FCP and HOMA-IR in T2DM patients were significantly increased and the ISI levels were significantly decreased (*P* < 0.05). The levels of HbA1c, CTGF, UACR, and miR-154-5p increased successively in the M1-3 groups while ADPN, P1NP, β-CTx, and OC decreased significantly (*P* < 0.05).

**Table 2:** Levels of serum miR-154-5p and clinical characteristic in male T2DM groups with different UACR.

	**M0**	**M1**	**M2**	**M3**	***F/*χ*^**2**^***	***P-*values**
*N*	141	138	127	109	–	–
Age (years)	54.09 ± 10.79	54.05 ± 10.69	52.17 ± 9.82	55.19 ± 9.02^c^	1.8258	0.1414
Course (months)	0 (0–0)	91 (84–99)^a^	92 (83–97)^a^	93 (85–100)^a^	314.2062	<0.0001
BMI (kg/m^2^)	25.00 ± 2.95	24.97 ± 2.97	24.44 ± 2.74	24.66 ± 2.62	1.1527	0.3273
WHR	0.92 ± 0.06	0.92 ± 0.06	0.91 ± 0.07	0.93 ± 0.07^c^	1.8038	0.1455
FPG (mmol/L)	5.61 ± 1.53	9.69 ± 2.48^a^	10.01 ± 2.56^a^	9.81 ± 2.47^a^	117.6565	<0.0001
FINS (mIU/L)	10.44 ± 3.14	12.40 ± 7.30^a^	11.92 ± 7.38	13.00 ± 8.06^a^	3.5073	0.0153
FCP (pmol/L)	6.81 ± 1.85	8.32 ± 2.34^a^	8.24 ± 2.44^a^	8.83 ± 2.58^a^	18.5787	<0.0001
ISI	−2.25 ± 0.51	−4.39 ± 1.23^a^	−4.45 ± 0.98^a^	−4.50 ± 1.08^a^	170.7250	<0.0001
HOMA-IR	2.56 ± 0.89	5.45 ± 3.75^a^	5.34 ± 3.80^a^	5.72 ± 4.03^a^	26.8978	<0.0001
HbA1c (%)	5.5 (5.2–5.8)	8.4 (7.7–9.3)^a^	9.0 (8.0–10.1)^a^^b^	9.7 (8.5–11.0)^a^^b^^c^	300.5268	<0.0001
HDL-C (mmol/L)	1.08 ± 0.26	1.08 ± 0.26	1.06 ± 0.23	1.05 ± 0.25	0.2803	0.8396
LDL-C (mmol/L)	3.36 ± 0.92	3.38 ± 0.91	3.09 ± 0.90^a^^b^	3.08 ± 1.02^a^^b^	3.9443	0.0084
TC (mmol/L)	4.99 ± 1.12	4.98 ± 1.12	5.09 ± 1.33	4.95 ± 1.30	0.3258	0.8067
TG (mmol/L)	2.24 ± 1.04	2.25 ± 1.04	2.16 ± 0.90	2.26 ± 1.02	0.2463	0.8640
ADPN (mg/L)	31.19 ± 2.74	27.46 ± 3.28^a^	23.57 ± 4.73^a^^b^	18.27 ± 6.60^a^^b^^c^	190.9144	<0.0001
25(OH)VD3(pg/ml)	7.96 (6.75–8.83)	7.97 (6.75–8.82)	7.81 (6.60–8.78)	7.89 (6.42–8.89)	0.1863	0.9798
PTH (pg/ml)	34.79 (31.08–38.03)	34.81 (31.11–38.03)	33.73 (30.82–36.69)	33.13 (30.16–36.63)	3.8989	0.2726
P1NP (pg/ml)	37.43 ± 9.89	37.27 ± 9.91	31.59 ± 8.91^a^^b^	25.54 ± 7.68^a^^b^^c^	45.0235	<0.0001
β-CTx (pg/ml)	410.46 ± 84.08	411.49 ± 84.50	376.31 ± 56.84^a^^b^	325.04 ± 38.08^a^^b^^c^	39.6529	<0.0001
Ca (mmol/L)	2.23 ± 0.15	2.23 ± 0.15	2.23 ± 0.14	2.24 ± 0.16	0.0910	0.9650
P (mmol/L)	1.16 ± 0.19	1.16 ± 0.19	1.16 ± 0.20	1.18 ± 0.20	0.3396	0.7967
ALP (U/L)	70.89 ± 22.46	70.29 ± 22.16	72.39 ± 18.02	70.16 ± 19.59	0.3037	0.8227
OC (pg/ml)	18.01 ± 6.77	15.73 ± 5.05^a^	12.24 ± 3.03^a^^b^	10.14 ± 1.84^a^^b^^c^	69.2499	<0.0001
CTGF (pg/ml)	159.49 ± 32.80	209.83 ± 42.95^a^	254.70 ± 52.03^a^^b^	394.31 ± 59.38^a^^b^^c^	551.4301	<0.0001
UACR (mg/g)	14.10 (12.64–15.47)	14.11 (12.58–15.48)	106.31 (93.14–114.59)^a^^b^	1062.24 (524.06–1530.10)^a^^b^^c^	400.8755	<0.0001
miR-154-5p	0.32 (0.15–0.52)	0.35 (0.32–0.40)	0.59 (0.52–0.66)^a^^b^	0.82 (0.72–0.90)^a^^b^^c^	315.5174	<0.0001

*Data are expressed as the mean ± SD or the median (interquartile range). BMI, body mass index; WHR, waist hip rate; FPG, fasting plasma glucose; FINS, fasting insulin; FCP, fasting c-peptide; ISI, insulin sensitive index (ISI = –ln[FPG × FINS]); HOMA-IR, homeostatic model assessment of insulin resistance (HOMA-IR = FPG × FINS/22.5); HbA1c, glycated hemoglobin A1c; HDL-C, high-density lipoprotein cholesterol; LDL-C, low-density lipoprotein cholesterol; TC, total cholesterol; TG, triglyceride; ADPN, adiponectin; PTH, parathyroid hormone; P1NP, procollagen type 1 N-terminal propeptide; β-CTx, β-C-terminal telopeptide of type I collagen; Ca, calcium; P, phosphorus; ALP, alkaline phosphatase; OC, osteocalcin; CTGF, connective tissue growth factor; UACR, urinary albumin excretion rate. M0, the normal control male group; M1-3, the normal albuminuria, microalbuminuria, and large amount of albuminuria male groups*.

a *M1-3 vs. M0, P < 0.05*.

b *M3, M2 vs. M1, P < 0.05*.

c *M3 vs. M2, P < 0.05*.

### Serum miR-154-5p Levels and Clinical Characteristic in Post-menopausal Female T2DM Groups With Different UACR

Compared to the F0 group, course, FPG, FINS, FCP and HOMA-IR in T2DM patients were significantly increased and the ISI levels were significantly decreased (*P* < 0.05) in the T2DM female group. Moreover, HbA1c, CTGF, UACR, and miR-154-5p levels in the post-menopausal female T2DM patients increased successively in the F1-3 groups while the levels of ADPN, P1NP, β-CTx, and OC were reduced (*P* < 0.05, Table 3 and Figure 1C).

**Table 3:** Levels of serum miR-154-5p and clinical characteristic in post-menopausal female T2DM groups with different UACR.

	**F0**	**F1**	**F2**	**F3**	***F/*χ*^**2**^***	***P-*values**
*N*	139	135	125	104	–	–
Age (years)	64.68 ± 10.53	64.71 ± 10.56	63.46 ± 9.96	62.05 ± 11.34	1.6426	0.1786
Course (months)	0 (0–0)	91 (83–100)^a^	93 (84–101)^a^	93 (86–100)^a^	308.8528	<0.0001
BMI (kg/m^2^)	24.35 ± 2.60	24.43 ± 2.59	24.78 ± 2.62	24.77 ± 2.63	0.9131	0.4344
WHR	0.93 ± 0.06	0.93 ± 0.06	0.92 ± 0.06	0.93 ± 0.06	0.8408	0.4720
FPG (mmol/L)	5.31 ± 1.66	8.53 ± 2.29^a^	8.52 ± 2.11^a^	8.65 ± 2.52^a^	77.5714	<0.0001
FINS (mIU/L)	9.61 ± 2.82	12.04 ± 7.20^a^	13.21 ± 7.05^a^	12.86 ± 7.29^a^	8.8203	<0.0001
FCP (pmol/L)	7.29 ± 2.03	8.79 ± 2.59^a^	8.72 ± 2.55^a^	8.38 ± 2.50^a^	11.2033	<0.0001
ISI	−2.24 ± 0.46	−4.29 ± 1.01^a^	−4.50 ± 0.78^a^	−4.39 ± 1.00^a^	224.4831	<0.0001
HOMA-IR	2.26 ± 0.93	4.56 ± 3.01^a^	5.10 ± 3.30^a^	4.95 ± 3.28^a^	30.5453	<0.0001
HbA1c (%)	4.6 (4.4–4.8)	7.5 (7.0–7.9)^a^	8.2 (7.3–8.7)^a^^b^	9.0 (8.1–10.0)^a^^b^^c^	82.9865	<0.0001
HDL-C (mmol/L)	1.10 ± 0.25	1.10 ± 0.25	1.09 ± 0.27	1.03 ± 0.26^a^^b^	355.2069	<0.0001
LDL-C (mmol/L)	3.13 ± 0.88	3.12 ± 0.89	3.24 ± 0.89	3.35 ± 0.91	1.6360	0.1801
TC (mmol/L)	5.04 ± 1.31	5.04 ± 1.30	4.88 ± 1.19	4.97 ± 1.11	0.4628	0.7084
TG (mmol/L)	2.18 ± 0.92	2.19 ± 0.93	2.03 ± 0.94	2.04 ± 1.00	1.0249	0.3812
ADPN (mg/L)	38.73 ± 3.71	34.23 ± 2.67^a^	30.03 ± 3.32^a^^b^	24.36 ± 5.09^a^^b^^c^	324.7018	<0.0001
25(OH)VD3(pg/ml)	8.02 (6.66–9.12)	7.93 (6.66–9.02)	7.48 (6.48–9.17)	8.10 (7.22–9.02)	3.3691	0.3381
PTH (pg/ml)	33.81 (30.51–37.12)	33.78 (30.51–37.24)	33.57 (30.10–35.97)	33.84 (30.25–36.96)	1.1314	0.7695
P1NP (pg/ml)	52.62 ± 15.85	52.27 ± 15.58	46.25 ± 13.04^a^^b^	41.34 ± 11.08^a^^b^^c^	16.8790	<0.0001
β-CTx (pg/ml)	571.07 ± 123.12	571.88 ± 123.39	515.35 ± 107.75^a^^b^	414.29 ± 104.81^a^^b^^c^	46.6004	<0.0001
Ca (mmol/L)	2.23 ± 0.15	2.23 ± 0.15	2.23 ± 0.15	2.23 ± 0.14	0.0340	0.9916
P (mmol/L)	1.19 ± 0.19	1.20 ± 0.18	1.16 ± 0.18	1.18 ± 0.21	0.7094	0.5467
ALP (U/L)	69.74 ± 20.03	69.51 ± 20.16	68.11 ± 18.29	71.10 ± 18.86	0.4561	0.7131
OC (pg/ml)	26.55 ± 9.03	21.26 ± 8.07^a^	17.89 ± 5.90^a^^b^	13.10 ± 5.01^a^^b^^c^	72.1206	<0.0001
CTGF (pg/ml)	168.82 ± 37.79	223.89 ± 79.06^a^	380.54 ± 72.94^a^^b^	465.46 ± 76.01^a^^b^^c^	496.1420	<0.0001
UACR (mg/g)	16.08 (14.34–17.75)	16.10 (14.43–17.75)	101.82 (90.85–111.36)^a^^b^	1246.65 (656.44–1723.61)^a^^b^^c^	407.8570	<0.0001
miR-154-5p	0.63 (0.30–1.05)	0.64 (0.56–0.75)	0.81 (0.69–0.97)^a^^b^	0.94 (0.78–1.09)^a^^b^^c^	82.9865	<0.0001

*Data are expressed as the mean ± SD or the median (interquartile range). BMI, body mass index; WHR, waist hip rate; FPG, fasting plasma glucose; FINS, fasting insulin; FCP, fasting c-peptide; ISI, insulin sensitive index (ISI = –ln[FPG × FINS]); HOMA-IR, homeostatic model assessment of insulin resistance (HOMA-IR = FPG × FINS/22.5); HbA1c, glycated hemoglobin A1c; HDL-C, high-density lipoprotein cholesterol; LDL-C, low-density lipoprotein cholesterol; TC, total cholesterol; TG, triglyceride; ADPN, adiponectin; PTH, parathyroid hormone; P1NP, procollagen type 1 N-terminal propeptide; β-CTx, β-C-terminal telopeptide of type I collagen; Ca, calcium; P, phosphorus; ALP, alkaline phosphatase; OC, osteocalcin; CTGF, connective tissue growth factor; UACR, urinary albumin excretion rate. F0, the normal control post-menopausal female group; F1-3, the normal albuminuria, microalbuminuria, and large amount of albuminuria post-menopausal female groups*.

a *F1-3 vs. F0, P < 0.05*.

b *F3, F2 vs. F1, P < 0.05*.

c *F3 vs. F2, P < 0.05*.

### Correlation Between OC, UACR, and Other Clinical Parameters in Male and Post-menopausal Female T2DM Patients

**Figure 2:**
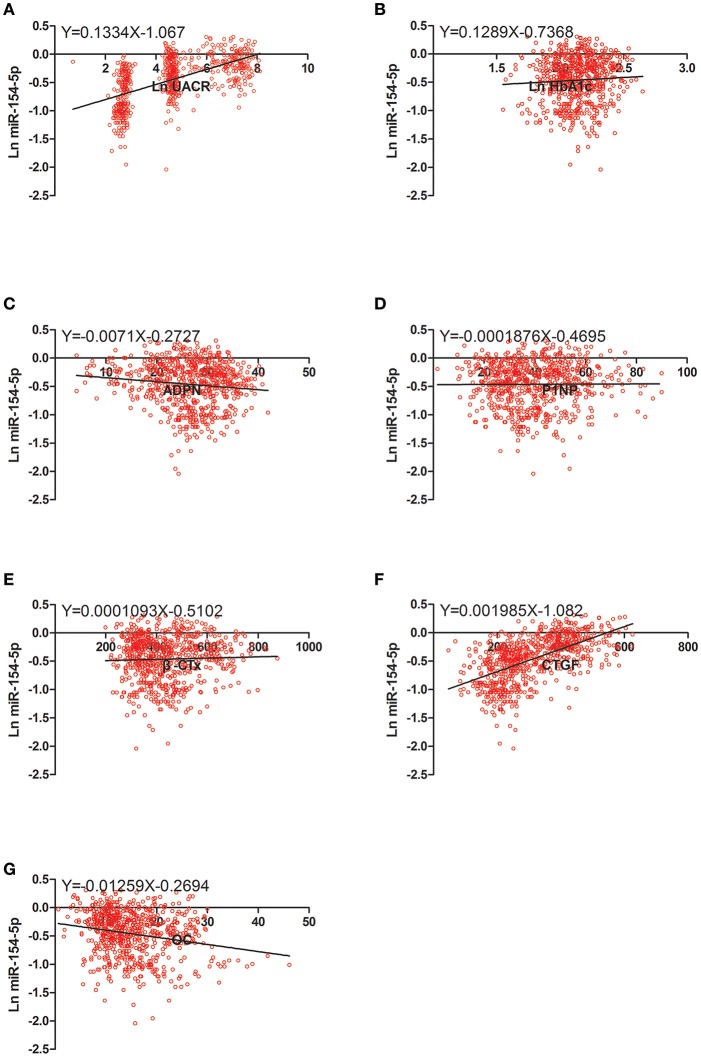
Scatter dot plots between Ln miR-154-5p and **(A)** Ln UACR, **(B)** Ln HbA1c, **(C)** ADPN, **(D)** P1NP, **(E)** β-CTx, **(F)** CTGF, **(G)** OC. UACR, urinary albumin excretion rate; HbA1c, glycated hemoglobin A1c; ADPN, adiponectin; P1NP, procollagen type 1 N-terminal propeptide; β-CTx, β-C-terminal telopeptide of type I collagen; CTGF, connective tissue growth factor; OC, osteocalcin.

**Figure 3:**
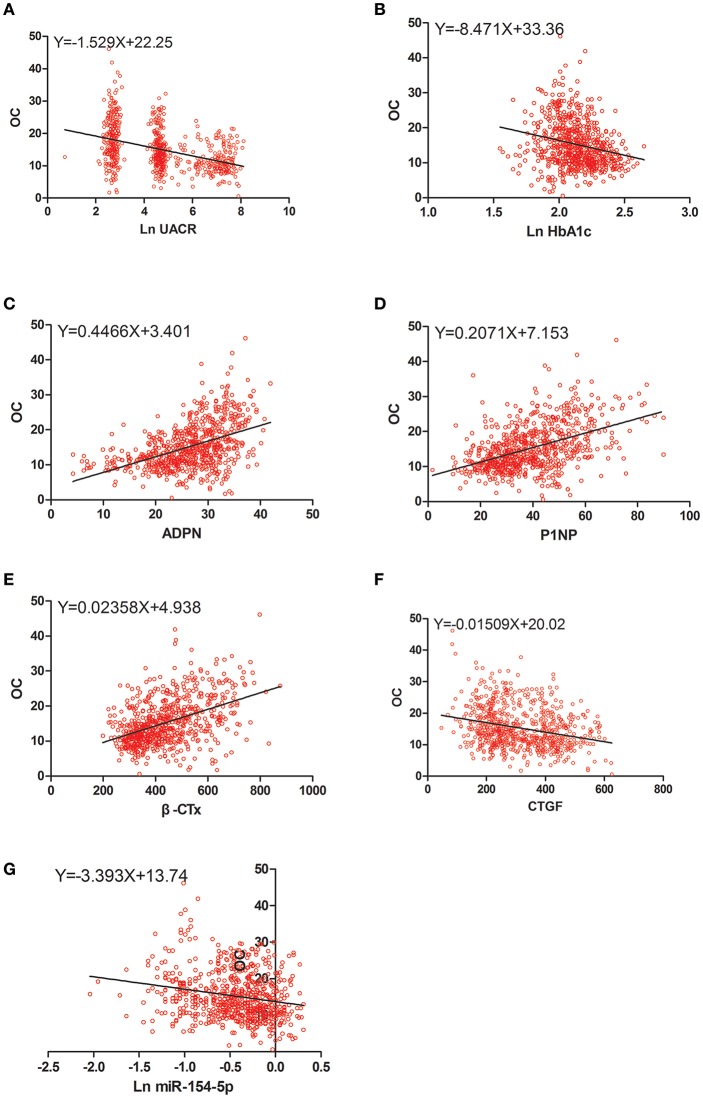
Scatter dot plots between OC and **(A)** Ln UACR, **(B)** Ln HbA1c, **(C)** ADPN, **(D)** P1NP, **(E)** β-CTx, **(F)** CTGF, **(G)** Ln miR-154-5p. UACR, urinary albumin excretion rate; HbA1c, glycated hemoglobin A1c; ADPN, adiponectin; P1NP, procollagen type 1 N-terminal propeptide; β-CTx, β-C-terminal telopeptide of type I collagen; CTGF, connective tissue growth factor; OC, osteocalcin.

**Figure 4:**
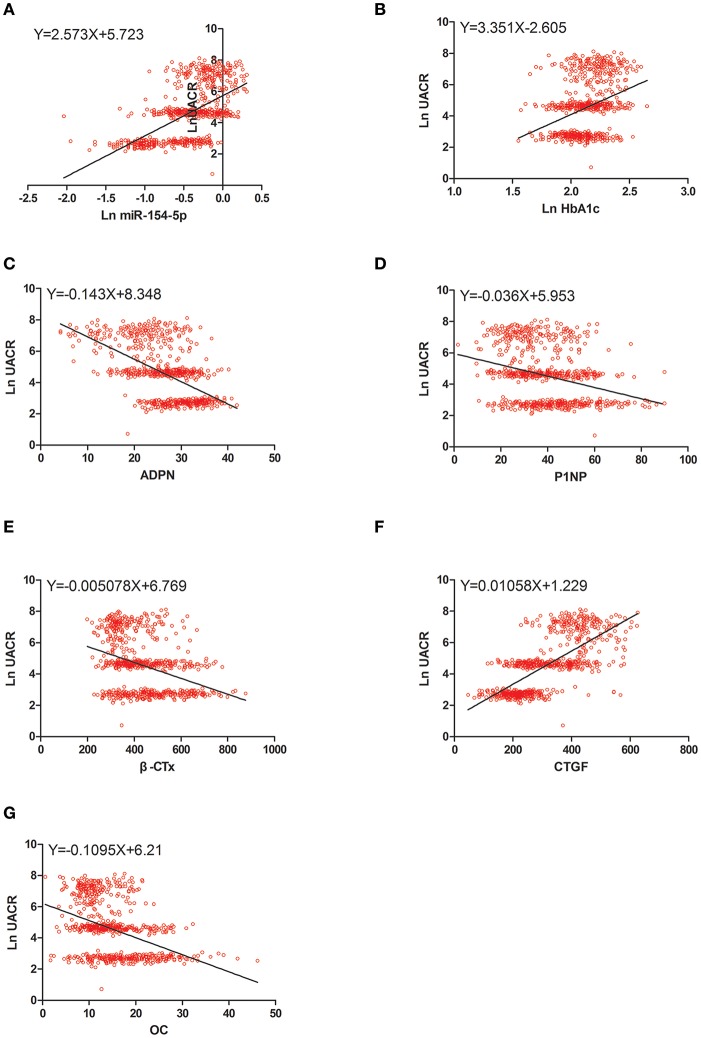
Scatter dot plots between Ln UACR and **(A)** Ln miR-154-5p, **(B)** Ln HbA1c, **(C)** ADPN, **(D)** P1NP, **(E)** β-CTx, **(F)** CTGF, **(G)** OC. UACR, urinary albumin excretion rate; HbA1c, glycated hemoglobin A1c; ADPN, adiponectin; P1NP, procollagen type 1 N-terminal propeptide; β-CTx, β-C-terminal telopeptide of type I collagen; CTGF, connective tissue growth factor; OC, osteocalcin.

Table 4 and Figures 2–4 shows the correlation analysis in all the groups of T2DM patients. Ln UACR was found to be positively correlated with CTGF, Ln HbA1c and Ln miR-154-5p and negatively correlated with ADPN, P1NP, β-CTx, and OC (*P* < 0.05). In contrast, OC showed a positive correlation with ADPN, P1NP, and β-CTx and negative correlation with Ln HbA1c, CTGF, Ln UACR, and Ln miR-154-5p (*P* < 0.05).

**Table 4:** Correlation between OC, UACR, Ln miR-154-5p, and other clinical parameters in male and post-menopausal female T2DM patients.

		**All**			**Male**			**Post-menopausal female**		
		**Ln miR-154-5p**	**OC**	**Ln UACR**	**Ln miR-154-5p**	**OC**	**Ln UACR**	**Ln miR-154-5p**	**OC**	**Ln UACR**
OC	*r*	−0.2070	1.0000	−0.4090	−0.5050	1.0000	−0.5090	−0.4870	1.0000	−0.4460
	*P*	<0.0001	–	<0.0001	<0.0001	–	<0.0001	<0.0001	–	<0.0001
ADPN	*r*	−0.1200	0.4590	−0.5500	−0.4790	0.3560	−0.5890	−0.3810	0.3610	−0.7200
	*P*	0.0011	<0.0001	<0.0001	<0.0001	<0.0001	<0.0001	<0.0001	<0.0001	<0.0001
CTGF	*r*	0.5790	−0.2680	0.7030	0.6430	−0.4330	0.7330	0.4490	−0.4290	0.7360
	*P*	<0.0001	<0.0001	<0.0001	<0.0001	<0.0001	<0.0001	<0.0001	<0.0001	<0.0001
P1NP	*r*	0.0069	0.4620	−0.3000	−0.4440	0.3710	−0.4650	−0.1980	0.3230	−0.3140
	*P*	0.8521	<0.0001	<0.0001	<0.0001	<0.0001	<0.0001	<0.0001	<0.0001	<0.0001
β-CTx	*r*	0.0344	0.4520	−0.3640	−0.4180	0.3380	−0.4470	−0.2150	0.3140	−0.4810
	*P*	0.3508	<0.0001	<0.0001	<0.0001	<0.0001	<0.0001	<0.0001	<0.0001	<0.0001
Ln UACR	*r*	0.5860	−0.4090	1.0000	0.7870	−0.5090	1.0000	0.4560	−0.4460	1.0000
	*P*	<0.0001	<0.0001	–	<0.0001	<0.0001	–	<0.0001	<0.0001	–
Ln miR-154-5p	*r*	1.0000	−0.2070	0.5860	1.0000	−0.5050	0.7870	1.0000	−0.4870	0.4560
	*P*	–	<0.0001	<0.0001	–	<0.0001	<0.0001	–	<0.0001	<0.0001
Ln HbA1c	*r*	0.0570	−0.2280	0.3370	0.2270	−0.1330	0.2530	0.1670	−0.1740	0.5090
	*P*	0.1220	<0.0001	<0.0001	<0.0001	0.0101	<0.0001	0.0014	0.0009	<0.0001

*HbA1c, glycated hemoglobin A1c; ADPN, adiponectin; P1NP, procollagen type 1 N-terminal propeptide; β-CTx, β-C-terminal telopeptide of type I collagen; CTGF, connective tissue growth factor; OC, osteocalcin; UACR, urinary albumin excretion rate*.

Moreover, Ln miR-154-5p was positively correlated with Ln HbA1c, CTGF, and Ln UACR and negatively correlated with ADPN and OC (*P* < 0.05). However, Ln miR-154-5p was negatively correlated with P1NP and β-CTx of single gender groups (*P* < 0.05), while no significant correlation was observed among two genders (*P* > 0.05).

### Ridge Regression Analysis of OC, UACR Levels, and Clinical Parameters in T2DM Patients

Multiple regression analysis was conducted by taking the indicators significantly related to Ln HbA1c, ADPN, P1NP, β-CTx, CTGF, OC, Ln UACR, and Ln miR-154-5p as independent variables and Ln UACR, OC as dependent variables. Exploratory multiple regression analysis showed that there was a significant correlation between the respective variables. As common linear regression analysis revealed severe colinearity (the colinearity diagnosis found that the maximum conditional index>30, variance expansion factor>10, variance component>0.5), we adopted a ridge regression analysis to address this problem.

**Figure 5:**
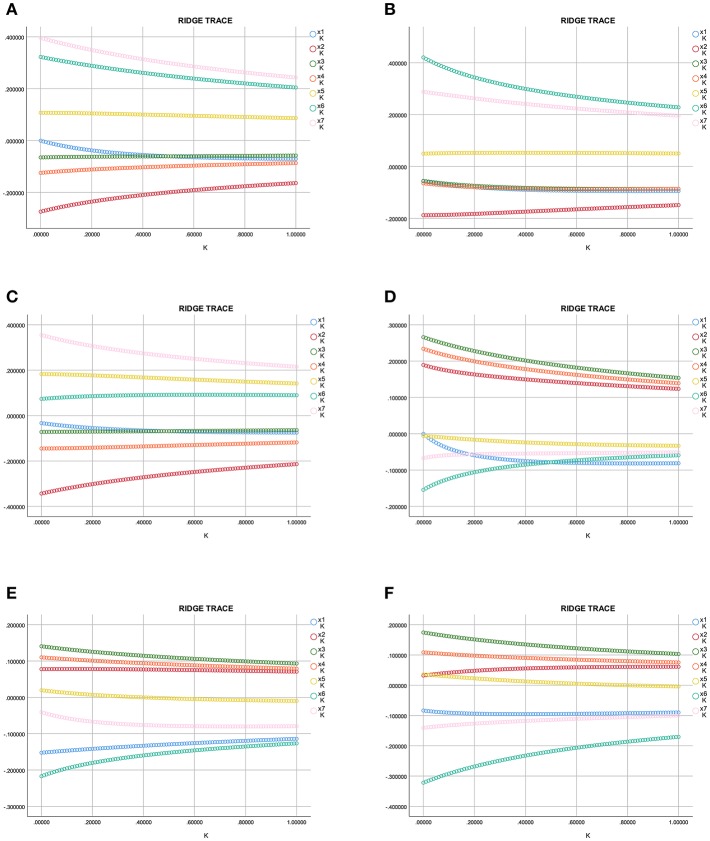
Ridge trace curve of the association between Ln UACR, OC, and the clinical parameters. **(A–C)** y = Ln UACR as dependent variables in all **(A)**, male **(B)**, and post-menopausal female **(C)** patients, and x1–x7 = OC, ADPN, P1NP, β-CTx, Ln HbA1c, Ln miR-154-5p, and CTGF as the independent variables, respectively; **(D–F)** y = OC as dependent variables in all **(D)**, male **(E)**, and post-menopausal female **(F)** patients, and x1–x7 = Ln UACR, ADPN, P1NP, β-CTx, Ln HbA1c, Ln miR-154-5p, and CTGF as the independent variables, respectively. UACR, urinary albumin excretion rate; HbA1c, glycated hemoglobin A1c; ADPN, adiponectin; P1NP, procollagen type 1 N-terminal propeptide; β-CTx, β-C-terminal telopeptide of type I collagen; CTGF, connective tissue growth factor; OC, osteocalcin.

The ridge trace shown in Figure 5 (x1–x7 = OC, ADPN, P1NP, β-CTx, Ln HbA1c, Ln miR-154-5p, and CTGF, respectively) with Ln UACR as the dependent variable in all, men and post-menopausal women T2DM patients indicated that when the ridge parameter was set at *k* = 0.4, standardized regression coefficients of each independent variable tended to be stable. Results showed that Ln UACR was affected by OC, ADPN, P1NP, β-CTx, CTGF, Ln HbA1c, and Ln miR-154-5p in all the three groups (*P* < 0.05, Table 5). Combined with the regression coefficients, we concluded that an increase in CTGF, Ln HbA1c, and Ln miR-154-5p resulted in an increase in Ln UACR, while an increase in ADPN, P1NP, β-CTx, and OC resulted in a decrease in Ln UACR.

**Table 5:** Ridge regression analysis of OC, UACR, and clinical parameters in male and post-menopausal female T2DM patients.

	**All**				**Male**				**Post-menopausal female**			
	**OC**		**Ln UACR**		**OC**		**Ln UACR**		**OC**		**Ln UACR**	
	***β***	***P***	***β***	***P***	***β***	***P***	***β***	***P***	***β***	***P***	***β***	***P***
Constant	7.2693	0.0004	4.0592	<0.0001	9.3882	0.0000	5.7971	<0.0001	12.0612	0.0004	3.4068	<0.0001
Ln HbA1c	−0.8903	0.2675	0.9988	<0.0001	−0.0027	0.9970	0.4879	0.0067	0.2707	0.8442	2.0205	<0.0001
ADPN	0.1455	<0.0001	−0.0545	<0.0001	0.0541	0.0166	−0.0490	<0.0001	0.0799	0.0237	−0.0865	<0.0001
P1NP	0.0903	<0.0001	−0.0073	0.0000	0.0490	0.0004	−0.0141	0.0000	0.0630	0.0000	−0.0084	0.0006
β-CTx	0.0093	<0.0001	−0.0014	<0.0001	0.0056	0.0033	−0.0020	0.0000	0.0048	0.0019	−0.0018	<0.0001
CTGF	−0.0031	0.0107	0.0047	<0.0001	−0.0036	0.0169	0.0045	<0.0001	−0.0065	0.0000	0.0038	<0.0001
Ln UACR	−0.2810	0.0002	–	–	−0.3328	0.0000	–	–	−0.4001	0.0001	–	–
OC	–	–	−0.0148	0.0002	–	–	−0.0358	0.0000	–	–	−0.0155	0.0014
Ln miR-154-5p	−1.3982	0.0001	1.1479	<0.0001	−1.7380	<0.0001	1.3029	<0.0001	−5.1221	<0.0001	0.5278	0.0000

*HbA1c, glycated hemoglobin A1c; ADPN, adiponectin; P1NP, procollagen type 1 N-terminal propeptide; β-CTx, β-C-terminal telopeptide of type I collagen; CTGF, connective tissue growth factor; OC, osteocalcin; UACR, urinary albumin excretion rate*.

The ridge trace was performed with Ln UACR, ADPN, P1NP, β-CTx, Ln HbA1c, Ln miR-154-5p, and CTGF as independent variables and OC as a dependent variable. Parameter estimation of the ridge regression was performed with *k* = 0.4 (*k* = 0.6 in Figure 5F). Using these settings, we found that OC was affected by ADPN, P1NP, β-CTx, CTGF, Ln UACR, and Ln miR-154-5p (*P* < 0.05, Table 5), but was not associated with Ln HbA1c (*P* > 0.05). Combined with the regression coefficients, it can be concluded that ADPN, P1NP, and β-CTx were positively correlated with OC while CTGF, Ln UACR and Ln miR-154-5p were negatively correlative with OC.

## Discussion

Mir-154 is a recently discovered microRNA with protective effect on cancer as demonstrated by two miRNA sequences (37, 38), miR-154-5p and miR-154-3p (39–41). Only a few studies have reported the role of miR-154-3p in cancer, and the exact target mRNA and molecular function of miR-154-3p remain unexplored (42, 43). The other mature sequence in human serum, miR-154-5p, is localized to the chromosome 14q32 microRNA with multiple binding elements and is involved in several diseases related to fibrosis (25, 26, 41, 44, 45). As chronic renal diseases can lead to proteinuria through renal fibrosis (27), miR-154-5p may affect the production of urinary protein.

A similar fundamental study documented that overexpression of miR-154-5p inhibited osteogenic differentiation of adipose-derived mesenchymal stem cells by suppressing expression of osteogenic marker gene and matrix mineralization. In contrast, inhibition of endogenous miR-154-5p promoted osteogenic differentiation (24).These findings indicate a negative regulatory role of miR-154-5p on osteogenic differentiation and warrant further investigation.

Based on the previous studies, we propose the hypothesis that serum miR-154-5p levels may be related to the mechanism of fibrosis in DKD and osteogenic differentiation of DOP in T2DM patients.

UACR is recommended as one of the basic classification standard of DKD by American Diabetes Association (ADA) and Kidney Disease: Improving Global Outcomes (KDIGO) (46, 47). Serum OC is a marker of bone formation and regeneration as well as an important indicator of bone conversion in DOP, which has been observed to correlate with microRNAs in post-menopausal females (8, 48, 49). To test our hypothesis, we investigated the expression levels of serum miR-154-5p and OC in men and post-menopausal women T2DM patients with different UACR, and analyzed correlation between these parameters. To clarify the effect of miR-154-5p in T2DM patients, we performed a cross-sectional cohort study and detected serum miR-154-5p and other glycolipid metabolic pathways, bone metabolism, urine albumin, and other biochemical indicators.

We observed significant differences in the levels of miR-154-5p, OC, and UACR between male and post-menopausal female patients groups and between the normal control groups and the T2DM groups with different UACR levels. In groups of single-sex T2DM patients, miR-154-5p increased, while OC decreased successively with UACR. Serum miR-154-5p was positively correlated with UACR and negatively correlated with OC. Collectively, our data suggest that circulating miR-154-5p may be involved in the pathological process of glycolipid metabolism, bone metabolism, and proteinuria.

There are two types of cells responsible for bone homeostasis, osteoblasts for secreting new bone, and osteoclasts for breaking bone down. The close cooperation between these cells determines the structure of bones in addition to supplying adequate of calcium. P1NP and ALP are markers of bone formation and β-CTx, a segment of collagen degradation excreted by the kidney during bone remodeling, is a marker of bone resorption. β-CTx is also one of the important clinical parameters utilized in the diagnosis and treatment of osteoporosis (9). As a sign of bone formation and regeneration, serum OC has been proven to be closely related to the above two processes in previous studies (8). 25 (OH) VD3 can promote the absorption of calcium and phosphorus in the intestinal tract and the re-absorption of calcium by renal tubules. PTH can strengthen osteolysis, enhance reabsorption of calcium by renal tubules, mobilize calcium into the blood, and increase blood calcium. All of these can increase the concentration of Ca and P in the blood and promote bone calcification (8, 9). In addition, all these biomarkers can affect the whole process of bone metabolism.

To further confirm the correlation between miR-154-5p and bone metabolism, we detected the serum levels of the bone metabolism related indicators (P1NP, β-CTx, 25[OH] VD3, PTH, ALP, Ca, and P) and found that P1NP and β-CTx were significantly increased in post-menopausal female compared to the male group. However, no significant difference was observed in the other bone metabolism factors (25[OH] VD3, PTH, ALP, Ca, and P) between the two groups. Compared to the normal control group, P1NP and β-CTx decreased successively with UACR in T2DM men and post-menopausal women. Pearson correlation analysis showed that miR-154-5p was significantly negatively correlated with P1NP and β-CTx in males and post-menopausal females with T2DM suggesting that miR-154-5p may be involved in the metabolic process of bone formation and absorption.

Our results also showed that serum miR-154-5p and HbA1c increased with the rise of UACR. Serum miR-154-5p and HbA1c were positively correlated with UACR indicating that miR-154-5p may be associated with chronic elevated blood glucose levels, thus involved in the pathological changes in DKD. However, the pathological mechanisms of the changes in urine protein caused by miR-154-5p remain unknown. CTGF is thought to regulate the progression of fibrosis in almost all diseases especially in renal tissues (50). Our data showed that CTGF increased with UACR and was positively correlated with UACR, providing supplementary evidence for the relationship between urinary protein changes and renal fibrosis. Further, we measured the levels of serum fibrosis factor CTGF and used correlation analysis to examine a possible relationship between miR-154-5p and CTGF. It was found that Ln miR-154-5p positively correlated with CTGF indicating that miR-154-5p is involved in the pathological process of renal injury by regulating fibrosis factors and causes the continuous increase of urinary protein.

Our study also found that OC, P1NP, and β-CTx were significantly reduced in male and post-menopausal female patients with different UACR, but there was no significant correlation with other bone metabolism indicators, consistent with previous literature that early chronic kidney disease can cause abnormal bone mineral metabolism and appearance of abnormal bone metabolic markers such as OC, P1NP, and β-CTx (14, 15). In addition, the levels of ADPN were significantly reduced and UACR was found to be negatively correlated with ADPN, an insulin–sensitizing hormone and blood lipid regulator, but not significantly correlated with other blood lipid indicators revealing a relationship between UACR and lipid metabolism, or potential associations with early metabolic processes such as insulin resistance and coronary atherosclerosis.

Our study showed that OC, P1NP, and β-CTx were significantly increased in post-menopausal women presenting a high bone transition state. OC positively correlated with P1NP and β-CTx adding to the evidence that OC is closely related to the osteogenesis process of bone formation and resorption. OC, encoded by bone gamma-carboxyglutamic acid-containing protein (BGLAP) gene in humans, is a specific protein solely secreted by osteoblasts in the general circulatory system during osteogenesis (51). As a hormone, OC plays a role in the metabolic regulation, bone mineralization, calcium ion homeostasis, is pro- osteoblastic, and involved in bone-building process.

OC can also cause islet beta cells to release more insulin and direct fat cells to release the hormone adiponectin thus increasing sensitivity to insulin (8). Clinical trials have reported that low OC levels were associated with metabolic syndrome, glucose, and lipid metabolism (10–13) suggesting that osteocalcin may be involved in the interactive relationship between bone and islet, adipose tissues. Studies investigating the relationship between OC and glucolipid metabolism have yielded conflicting data and the underlying mechanisms remain unclear (11, 52–56). Similar observations in our research have showed OC positively correlated with ADPN and negatively correlated with HbA1c, but no significant differences were observed between OC and other glucose and lipid metabolism indicators (FPG, FINS, FCP, ISI, HOMA-IR, and blood lipids) which is consistent with the clinical evidence and our previous study (57, 58). The relationship between OC, blood glucose and insulin may be interrupted by no limit of hypoglycemic drugs, and unmeasured undercarboxylated OC (ucOC), the active form of OC (59). In addition, different race selection, experimental design, index measurement, and analysis may account for these differences.

We adopted men and post-menopausal women as the research volunteers to avoid the effect of estrogen on metabolism, resulting in the age-mismatched phenomenon in different genders. Age, WHR, ADPN, P1NP, β-CTx, OC, CTGF, UACR, and mir-154-5p were significantly increased in post-menopausal women, while FPG, HOMA-IR and HbA1c decreased compared with the male group. However, according to the results of correlation analysis, glucolipid, and bone metabolic factors had no significant correlation with age, thus excluding significant differences resulting from age differences, revealing that changes in metabolic factors are caused by DOP rather than senile osteoporosis.

However, there are several limitations of this study: (1) as a cross-sectional cohort study, this study only indicates a correlation between miR-154-5p, OC, and UACR. There is a need for a follow-up and long-term observational trials and survival analyses to validate these observations. (2) Subjects with pre-diabetes were not included and the quality control of hypoglycemic drugs was not conducted. (3) We used ridge regression to solve the problem of collinearity in multiple regression which means a modified analysis of least squares estimation by abandoning the unbiasedness of least squares. The regression coefficient is obtained at the cost of partial information loss and accuracy reduction, however, the fitting of pathological data is stronger than that of least squares.

In summary, our study speculates that serum miR-154-5p levels is positively correlated with HbA1c, UACR, and CTGF and negatively correlated with ADPN, OC, P1NP, and β-CTx. These findings indicate that miR-154-5p may participate in the pathological process of glucolipid, bone metabolism, and renal fibrosis, which may further contribute to novel target development for DKD and DOP. However, the specific mechanisms of miR-154-5p remain elusive and still present a challenge to medical research field.

## Ethics Statement

This study was approved by the Ethics Committee of the First Affiliated Hospital of China Medical University. All procedures were performed in accordance with the ethical standards as mentioned in the 1964 Declaration of Helsinki and its later amendments or comparable ethical standards.

## Statement of Informed Consent

Informed consent was obtained from all participants included in the study.

## Author Contributions

HR and QW designed the experiments and wrote the manuscript. HR, XM, YS, MY, and JH carried out the experiments and collected the cases and data. HR analyzed the experimental results.

### Conflict of Interest Statement

The authors declare that the research was conducted in the absence of any commercial or financial relationships that could be construed as a potential conflict of interest.
